# Increasing Compliance with Mass Drug Administration Programs for Lymphatic Filariasis in India through Education and Lymphedema Management Programs

**DOI:** 10.1371/journal.pntd.0000728

**Published:** 2010-06-29

**Authors:** Paul T. Cantey, Jonathan Rout, Grace Rao, John Williamson, LeAnne M. Fox

**Affiliations:** 1 Epidemic Intelligence Service, Office of Workforce and Career Development, Centers for Disease Control and Prevention, Atlanta, Georgia, United States of America; 2 Division of Parasitic Diseases, National Center for Zoonotic, Vector-borne and Enteric Diseases, Centers for Disease Control and Prevention, Atlanta, Georgia, United States of America; 3 Church's Auxiliary for Social Action, Bhubaneswar Sector, Bhubaneswar, India; University of Kelaniya, Sri Lanka

## Abstract

**Background:**

Nearly 45% of people living at risk for lymphatic filariasis (LF) worldwide live in India. India has faced challenges obtaining the needed levels of compliance with its mass drug administration (MDA) program to interrupt LF transmission, which utilizes diethylcarbamazine (DEC) or DEC plus albendazole. Previously identified predictors of and barriers to compliance with the MDA program were used to refine a pre-MDA educational campaign. The objectives of this study were to assess the impact of these refinements and of a lymphedema morbidity management program on MDA compliance.

**Methods/Principal Findings:**

A randomized, 30-cluster survey was performed in each of 3 areas: the community-based pre-MDA education plus community-based lymphedema management education (Com-MDA+LM) area, the community-based pre-MDA education (Com-MDA) area, and the Indian standard pre-MDA education (MDA-only) area. Compliance with the MDA program was 90.2% in Com-MDA+LM, 75.0% in Com-MDA, and 52.9% in the MDA-only areas (p<0.0001). Identified barriers to adherence included: 1) fear of side effects and 2) lack of recognition of one's personal benefit from adherence. Multivariable predictors of adherence amenable to educational intervention were: 1) knowing about the MDA in advance of its occurrence, 2) knowing everyone is at risk for LF, 3) knowing that the MDA was for LF, and 4) knowing at least one component of the lymphedema management techniques taught in the lymphedema management program.

**Conclusions/Significance:**

This study confirmed previously identified predictors of and barriers to compliance with India's MDA program for LF. More importantly, it showed that targeting these predictors and barriers in a timely and clear pre-MDA educational campaign can increase compliance with MDA programs, and it demonstrated, for the first time, that lymphedema management programs may also increase compliance with MDA programs.

## Author Summary

Global elimination of lymphatic filariasis requires giving drugs at least annually to populations who live at risk of becoming infected with the parasite. At least 80% of people at risk need to take the drugs annually for 5 or more years to stop transmission of the infection. People suffering from the long-term effects of infection, such as swollen legs, benefit from programs that teach self-care of their affected limbs. In this study, we assessed the impact of an educational campaign that, after addressing previously identified predictors of compliance, significantly improved drug compliance. The specific factors improving compliance included knowing about the drug distribution in advance, knowing that everyone is at risk for acquiring the infection, knowing that the drug distribution was for lymphatic filariasis prevention, and knowing at least one component of leg care. We also found that areas with programs to assist people with swollen legs had greater increases in compliance. This research provides evidence that program evaluation can be used to improve drug compliance. In addition, our work shows for the first time that programs to benefit people with swollen legs caused by lymphatic filariasis also increase the participation of people without disease in drug treatment programs.

## Introduction

There are 1.3 billion people living at risk of infection with the parasites that cause lymphatic filariasis (LF) and an estimated 40 million suffering from the long-term complications of the disease [1], [2]. In 2000, the Global Programme for Elimination of LF (GPELF) began its campaigns to interrupt transmission of the parasite using a strategy of annual mass drug administration (MDA) to those at risk and to control or prevent LF-related disability through morbidity management programs [3].

India's National Vector Borne Disease Control Programme has scaled up MDA to interrupt LF transmission over the past several years and recently began adding albendazole to diethylcarbamazine (DEC) therapy where available with the monumental goal of providing mass drug treatment to all 590 million Indians living at risk for infection [4]. Although the program has distributed sufficient quantities of DEC tablets, problems have remained with achieving sufficient levels of adherence to DEC regimens in many regions in India [5]–[10], including Orissa State [11], [12]. Published estimates reporting drug coverage are often more accurately characterized as estimates of drug distribution which overestimate the actual drug consumption or compliance with MDA of the population [6], [13]. Mathematical models suggest interrupting transmission is dependent on the baseline population prevalence of LF infection and on overall population compliance with MDA programs [14], [15]. The lower the compliance with the MDA and the higher the baseline prevalence of LF, the more rounds of MDA required to interrupt transmission. Ensuring maximal compliance is critical to programmatic success.

In some areas of India, the MDA program is restricted to tablet distribution, and issues such as drug adherence, drug side effects, and LF education of the populace are not comprehensively addressed [13]. For this reason, in 2007 the Church's Auxiliary for Social Action (CASA) partnered with the Indian Ministry of Health in Orissa State to enhance adherence to the DEC regimen. CASA developed a community-based educational campaign for the populace in three sub-districts in Khurda District of Orissa State. The campaign sought to increase awareness about the occurrence of the MDA, about the transmission and prevention of LF, about who should take DEC and the potential side effects, and about mosquito control. The message was distributed over a four-week period prior to the December 2007 MDA via radio and newspaper advertisements, street plays, leaflet distributions, broadcasting local songs incorporating health messages, posters, wall paintings, and village educational sessions. CASA partnered with the Centers for Disease Control and Prevention (CDC) to evaluate the effectiveness of their program. This evaluation found that adherence to the DEC regimen was less than 60% and failed to detect a significant impact of the community-based education campaign [16]. However, it identified barriers to and predictors of adherence. The major barriers were fear of side effects and a lack of recognition of the benefit of adherence. A model of predictors of adherence to the DEC regimen found that people who knew about the MDA in advance of its occurrence, people who knew that the MDA was for LF prevention, and people who knew that mosquitoes transmit LF were significantly more likely to adhere to the medication. The model also suggested that those who knew everyone was at risk for LF were also more likely to adhere, though this was not statistically significant. Other studies have found similar barriers and predictors using a variety of methodologies [5], [9], [11], [12], [17]–[21], though relatively few provided a quantitative measure of association [22], [23].

Based on these data, the CASA community-based educational message was refined to focus on these predictors and barriers. For the December 2008 MDA, CASA expanded its community-based educational campaign adding three new sub-districts to the original three in Khurda District. These new sub-districts received the same educational campaign described for the 2007 pre-MDA campaign, but the educational messages incorporated these refinements. Additionally, in early 2008, CASA initiated a lymphedema management program which included both a community-based education component for the entire populace and patient self-care component focused on foot and leg hygiene for affected individuals and their families. This program was initiated in the three original sub-districts only.

This evaluation was designed with the following objectives: 1) to assess the effectiveness of community-based LF education and community-based lymphedema management education in increasing compliance with the MDA program and 2) to validate the importance of predictors of and barriers to adherence to the DEC regimen identified in the previous evaluation.

## Methods

### Ethics Statement

The program was approved by the National Center for Zoonotic, Vector-borne, and Enteric Diseases (NCZVED) Human Subjects Committee at CDC, Atlanta, Georgia, USA, prior to the implementation of the survey. Permission for the survey was obtained from the Orissa State Department of Health and Family Welfare. Participants were asked to give their written consent prior to participation. For those unable to write, consent was documented by recording the person's fingerprint or marking the signature line with an ‘X’ and by countersignature of survey personnel. Consent procedures were approved by the Human Subjects Committee.

### Study Design

The 2008 MDA for Orissa occurred from December 28^th^ to 30^th^. The coverage survey occurred from February 19^th^ to 28^th^, 2009. A random 90-cluster sample design was utilized, with 30 villages selected in each of three areas: the community-based pre-MDA education plus community-based lymphedema management education (Com-MDA+LM) area, the community-based pre-MDA education (Com-MDA) area, and the Indian standard pre-MDA education (MDA-only) area. The Com-MDA+LM area included the three original sub-districts―Khurda, Balianta, and Balipatna―that received both the community-based pre-MDA educational campaign and the community-based lymphedema management program. The Com-MDA areas included the three new sub-districts―Bologarh, Begunia, and Jatni―that received only the pre-MDA educational campaign. The MDA-only area was composed of one sub-district―Banapur―which did not border any of the other six sub-districts and received only the standard Indian Ministry of Health MDA campaign. Villages were selected in each area using probability proportionate to size methodology [24], [25]. In villages with hamlets, which are areas of a village separate from the main village, probability proportionate to size methodology was used to determine whether the hamlet or the main village was sampled. Fifteen households were randomly selected using the Expanded Programme on Immunization (EPI) random walk methodology [24]. Two quantitative surveys were performed: a household (HH) survey, in which every member of the household was included, and a knowledge, attitudes, and practices (KAP) survey, in which one person in each household over the age of 17 was randomly chosen to participate. Replacement of non-responders was not permitted.

The survey was designed to detect a difference in drug adherence of 15% between the areas. Calculations were adjusted to allow for two 2-way comparisons and to account for a design effect of 12, based on the design effect found in the 2008 study [16]. Thus the study had 80% power to detect 15% difference with an alpha of 0.025 if 3,181 persons were enrolled in each study area.

### Analysis

Data were entered into EpiInfo v3.5.1 (Stone Mountain, GA) and analysis was performed in SAS v9.2 (Cary, NC). All results presented from the HH and KAP surveys were adjusted for stratification and clustering, except for tests for medians. All KAP survey results were also weighted by the size of the eligible population in the household. For differences between the three areas, dichotomous variables were evaluated using chi-square tests or Rao-Scott Likelihood Ratio Tests and continuous variables were evaluated using tests for medians. Multivariable logistic regression analysis of KAP data was performed to assess predictors of adherence to DEC. All predictors that were statistically significant (p≤0.05) in univariable analysis were included in the final model. Any demographic variable not found to be a univariable predictor of adherence but which differed across the three groups was included in the initial model. Interaction terms were created and removed by examining the Wald chi-squares for the individual components of the interaction terms. After evaluation of the interaction terms, demographic variables that were not found to be predictors of adherence were removed if removal did not change the adjusted odds ratios (OR) for the major predictors by at least 10% and if removal improved the precision of the estimates for the major predictors. The model was adjusted for weighting, clustering, and stratification.

## Results

### Household Survey

In Com-MDA+LM areas 449 (99.7%) households participated in the HH survey, in Com-MDA areas 427 (94.9%) participated, and in MDA-only areas 409 (90.9%) participated. There were 2949, 2863, and 2481 persons included in the survey, respectively. The groups were similar in age distribution (median 29 years, range 0.1 years–105 years), and sex distribution (52.0% male). Tablets were received by 2784 (94.4%), 2671 (93.3%) and 2105 (84.9%) persons, respectively (p<0.0001). There were three (0.1%), 67 (2.3%) and 49 (2.0%) persons eliminated from further analysis because they did not live in the respective area at the time of the MDA.

Adherence to the DEC regimen differed significantly between the three areas (Table 1), with 90.2% adherence in Com-MDA+LM areas, 75.0% adherence in Com-MDA areas, and 52.9% adherence in MDA-only areas. Among those who took DEC, 217 (3.6%) reported side effects, the most common of which was headache (125, 2.1%). All three groups reported similar rates of side effects (p = 0.2). No one required hospitalization for side effects. Persons who did not take DEC were asked why. All reasons provided by more than 5% of the population are shown in Table 1. The most common reason given in all areas was fear of side effects, though persons in the Com-MDA+LM area were the least likely to give this reason. Com-MDA+LM persons were more likely to state that they were sick at the time of the MDA, which is a legitimate contraindication in the Indian program.

**Table 1:** Household survey: Rates of adherence and non-adherence to a DEC regimen and reasons for non-adherence during the 2008 MDA, Khurda District, Orissa State.

Category	Com-MDA+LM^a^ n (%)	Com-MDA^b^ n (%)	MDA-only^c^ n (%)	p-value 1^d^	p-value 2^e^
Adhered to DEC	2658 (90.2)	2097 (75.0)	1285 (52.9)	<0.0001	<0.0001
Did not adhere to DEC	288 (9.8)	698 (25.0)	1146 (47.1)		
Top reasons for non-adherence:					
Fear of side effects	22 (17.6)	258 (45.3)	263 (32.3)	0.0008	0.07
Lack of trust of DEC	12 (9.6)	53 (9.3)	183 (22.5)	0.98	0.1
Not present for DEC distribution	22 (17.6)	136 (23.9)	48 (5.9)	0.4	<0.0001
Forgot to take DEC	5 (4.0)	22 (3.9)	136 (16.7)	0.95	<0.0001
DEC not needed/not sick	13 (10.4)	64 (11.2)	56 (6.9)	0.85	0.25
Sick when DEC given out	35 (28.0)	17 (3.0)	65 (8.0)	<0.0001	0.001

Note: A p-value ≤0.025 is considered statistically significant as a correction for multiple comparisons.

a Com-MDA+LM areas received community-based pre-MDA LF education and a community-based lymphedema management program.

b Com-MDA areas received community-based pre-MDA LF education.

c MDA-only areas received the Indian Ministry of Health MDA campaign.

d P-value 1 is for the comparison between Com-MDA+LM and Com-MDA.

e P-value 2 is for the comparison between Com-MDA and MDA-only.

### KAP Survey

In Com-MDA+LM areas 445 (98.9%) persons participated in the KAP survey, in Com-MDA areas 423 (94.0%) participated, and in MDA-only areas 401 (89.1%) participated. One Com-MDA+LM person was eliminated from the analysis because her answers could not be weighted. The demographic breakdown of KAP participants is shown in Table 2. There were statistically significant differences in the sex, age, caste, educational level, and literacy level distributions between the three groups. Although households reporting at least one household member with a swollen leg ranged from 9.7% to 18.6% to 23.5%, this difference was not statistically significant (p = 0.28 for overall comparison across the three groups).

**Table 2:** Demographics for KAP survey participants for the 2008 MDA, Khurda District, Orissa State.

Category	Com-MDA+LM^a^ n (%)	Com-MDA^b^ n (%)	MDA-only^c^ n (%)	p-value overall
Male sex	217 (48.3)	172 (42.2)	145 (35.4)	0.009
Age, years^d^	30 (14–105)	35 (17–80)	34 (18–80)	<0.0001
Caste:				0.02
General castes	185 (44.9)	217 (49.4)	89 (21.6)	
Backward castes	179 (40.1)	171 (43.2)	258 (67.4)	
Scheduled castes & tribes	81 (11.2)	35 (7.4)	52 (10.8)	
Education:				<0.0001
No schooling	6 (0.8)	33 (6.9)	85 (16.8)	
Grades 1 to 5	111 (24.1)	151 (34.1)	157 (38.9)	
Grades 6 to 10	247 (54.6)	187 (46.9)	106 (31.0)	
Grades 11 to 12	44 (10.7)	36 (8.8)	18 (4.7)	
Graduate and Post-graduate	36 (9.6)	16 (3.3)	32 (8.2)	
Literacy:				<0.0001
Reads well	363 (83.5)	289 (68.3)	180 (52.7)	
Reads with difficulty/not at all	79 (16.5)	134 (31.7)	201 (47.3)	
Household member with swollen leg	89 (23.5)	75 (18.6)	40 (9.7)	0.28

Note: P-values derived from the Rao-Scott Likelihood Ratio Test except where noted.

a Com-MDA+LM areas received community-based pre-MDA LF education and a community-based lymphedema management program.

b Com-MDA areas received community-based pre-MDA LF education.

c MDA-only areas received the Indian Ministry of Health MDA campaign.

d Median age (range), p-value for the difference in medians.

Participants who complied with the MDA program were asked why they took DEC. The most common reasons given were as follows: 1) to prevent LF (463, 48.0%), 2) because the MDA distributor told me to take DEC (344, 32.9%), and 3) because a family member told me to take DEC (211, 22.6%). Participants who did not take DEC were asked to specify why and what they would need to be told to change their minds. The top five reasons given were as follows: 1) fear of side effects (80, 30.3%), 2) lack of trust of DEC (45, 16.9%), 3) sick at the time of the MDA (29, 9.5%), 4) not at home when DEC was distributed (25, 9.2%), and 5) not sick and therefore DEC was not needed (25, 9%). They reported they would comply if convinced that taking DEC would help them (151, 52.3%), if convinced that taking DEC would help their family (53, 17.3%), or if taught to manage side effects (22, 9.8%).

Participants were asked questions about their knowledge of LF, MDA, and lymphedema management. Responses are shown in Table 3. Com-MDA+LM participants had greater knowledge than Com-MDA participants that LF is transmitted by mosquitoes, everyone is at risk for LF, and there are specific treatments for lymphedema such as leg exercises, leg washing, and leg elevation. Com-MDA participants were much more likely than MDA-only participants to know about the MDA in advance of its occurrence, mosquitoes transmit LF, and antibiotics can be used to help manage acute attacks. Com-MDA and MDA-only participants were equally likely to know everyone was at risk for LF.

**Table 3:** Knowledge about LF and MDA among KAP survey participants from the 2008 MDA, Khurda District, Orissa State.

	Com-MDA+LM^a^ n (%)	Com-MDA^b^ n (%)	MDA-only^c^ n (%)	p-value 1^d^	p-value 2^e^
Knowledge item:					
Knew about MDA in advance	425 (97.1)	387 (91.5)	257 (69.1)	0.03	<0.0001
Knew MDA was for LF	439 (98.8)	386 (91.8)	368 (92.4)	<0.0001	0.82
Knew mosquitoes transmit LF	426 (95.7)	356 (84.0)	269 (68.9)	<0.0001	0.001
Thought contaminated water transmits LF	12 (2.7)	45 (12.4)	22 (4.9)	<0.0001	0.01
Knew everyone at risk for LF	403 (90.8)	208 (47.3)	173 (44.6)	<0.0001	0.67
Thought old people at risk for LF	16 (4.3)	136 (34.3)	162 (41.9)	<0.0001	0.22
Lymphedema treatments:					
Antibiotics	211 (49.7)	153 (35.6)	41 (9.6)	0.05	<0.0001
No treatment	63 (14.9)	93 (25.3)	152 (37.6)	0.06	0.07
Leg exercises	128 (30.8)	19 (5.3)	6 (1.6)	<0.0001	0.04
Leg washing	98 (21.6)	22 (5.5)	9 (2.8)	<0.0001	0.14
Leg elevation	31 (8.4)	6 (1.3)	3 (0.8)	<0.0001	0.52
Any of the 3 leg care answers	201 (46.3)	43 (11.3)	16 (4.6)	<0.0001	0.005

Note: Percentages are weighted. P-values derived from the Rao-Scott Likelihood Ratio Test. P-values ≤0.025 are significant.

a Com-MDA+LM areas received community-based pre-MDA LF education and a community-based lymphedema management program.

b Com-MDA areas received community-based pre-MDA LF education.

c MDA-only areas received the Indian Ministry of Health MDA campaign.

d P-value 1 is for the comparison between Com-MDA+LM and Com-MDA.

e P-value 2 is for the comparison between Com-MDA and MDA-only.

Demographic and knowledge variables were examined to determine if they were univariable predictors of adherence to the DEC regimen. Results are shown in Table 4. Neither caste nor male sex was a univariable predictor. Some quartiles of age, having 11 to 12 years of education, reading well, and having a household member with lymphedema were found to be predictors. More importantly, five factors that could be addressed in educational campaigns were found to predict adherence. They are, in decreasing order of strength of association: knowing about the MDA in advance of its occurrence (OR = 8.1; 95% CI: 5.2–12.6), knowing the MDA was for LF (OR = 7.5; 95% CI: 4.3–12.9), knowing one or more components of lymphedema management (OR = 7.4; 95% CI: 3.8–14.6), knowing everyone was at risk for LF (OR = 3.7; 95% CI: 2.5–5.3), and knowing mosquitoes transmit LF (OR = 3.2; 95% CI: 2.2–4.7). Multivariable modeling was then performed. A statistically significant interaction between knowing about the MDA in advance of its occurrence and knowing everyone was at risk for LF was found and therefore was kept in the model. Male sex did not influence the model and was removed. Caste, literacy, and having a household member with lymphedema did not predict adherence in multivariable analysis. Some quartiles of age and multiple educational levels influenced adherence. Significant predictors, which could be addressed in an educational campaign, included knowing both about the MDA in advance and that everyone was at risk for LF (adjusted OR = 16.1; 95% CI: 8.8–29.3), knowing about the MDA in advance (adjusted OR = 4.8; 95% CI: 3.8–8.1), knowing everyone was at risk for LF (adjusted OR = 2.2; 95% CI 1.0–4.8; p = 0.04), knowing the MDA was for LF (adjusted OR = 3.3; 95% CI: 1.7–6.6), and knowing at least one component of lymphedema management self-care (adjusted OR = 3.3; 95% CI: 1.6–6.9).

**Table 4:** Univariable & multivariable analyses of predictors of adherence to a DEC regimen among KAP survey participants from the 2008 MDA, Khurda District, Orissa State.

Variables	Univariable analysis OR (95% CI)	Multivariable analysis OR (95% CI)
Demographic variables:		
Quartiles of age:		
Age ≤25 years	1.7 (1.1–2.8)	1.8 (1.0–3.4)^a^
Age >25 & ≤35 years	1.3 (0.8–2.0)	1.3 (0.8–2.2)
Age >35 & ≤45 years	1.9 (1.2–3.1)	2.0 (1.2–3.2)
Age >45 years	referent	referent
Male sex	1.1 (0.8–1.6)	…
Caste:		
General castes	referent	referent
Backward castes	0.9 (0.5–1.4)	1.0 (0.6–1.4)
Scheduled castes & tribes	1.1 (0.5–2.5)	2.1 (0.9–4.8)
Education:		
No school	0.6 (0.3–1.3)	3.5 (1.2–10.0)
Grades 1 to 5	1.3 (0.7–2.5)	3.2 (1.4–7.3)
Grades 6 to 10	1.7 (0.9–3.2)	2.0 (1.0–4.1)^a^
Grades 11 to 12	2.6 (1.1–6.2)	3.0 (1.0–8.7)^a^
Graduate or post-graduate	referent	referent
Literacy:		
Reads well	2.1 (1.4–3.0)	1.6 (0.9–2.6)
Reads with difficulty/not at all	referent	Referent
Household member with leg edema	2.0 (1.2–3.3)	1.4 (0.8–2.4)
Knowledge variables:		
Knew about MDA in advance	8.1 (5.2–12.6)	…
Knew the MDA was for LF	7.5 (4.3–12.9)	3.3 (1.7–6.6)
Knew about lymphedema management^b^	7.4 (3.8–14.6)	3.3 (1.6–6.9)
Knew everyone at risk for LF	3.7 (2.5–5.3)	…
Knew mosquitoes transmit LF	3.2 (2.2–4.7)	1.3 (0.8–2.1)
Interaction between knew about MDA in advance & knew everyone at risk for LF:		
Knew both	…	16.1 (8.8–29.3)
Only knew about MDA in advance	…	4.8 (3.8–8.1)
Only knew everyone at risk for LF	…	2.2 (1.0–4.8)^c^
Knew neither	…	Referent

a P-value = 0.05.

b Knew about lymphedema management means the participant could name at least one of the three components of lymphedema management: leg washing, leg elevation, or leg exercises.

c P-value = 0.04.

To further examine the impact of the lymphedema management programs on adherence, two sub-analyses were performed. In the first sub-analysis, the knowledge of univariable predictors of adherence was compared between those who knew at least one of the three components of lymphedema leg care and those who did not know any. Only persons in Com-MDA+LM and Com-MDA populations were included. As shown in Table 5, persons who knew at least one component of leg care had greater knowledge of all four of the univariable predictors of DEC adherence. The second sub-analysis drew from this same population. Multivariable analysis which included all predictors of adherence from the main model was performed among those who had a household member with leg swelling and among those who did not. Having knowledge of at least one component of leg care predicted increased adherence both among those who had a household member with leg swelling (adjusted OR = 11.1, 95% CI: 1.4–86.1), and among those without (adjusted OR = 5.1, 95% CI: 1.5–17.4).

**Table 5:** Knowledge of univariable predictors of adherence to a DEC regimen among KAP participants from Com-MDA+LM^a^ and Com-MDA^b^ areas: examining the difference in knowledge based on knowledge of lymphedema management^c^.

Knowledge item	Knew lymphedema management n (%)	Did not know lymphedema management n (%)	p-value^d^
Knew about MDA in advance	241 (99.5)	571 (92.4)	<0.0001
Knew MDA was for LF	239 (98.6)	586 (94.1)	<0.0001
Knew mosquitoes transmit LF	235 (96.6)	547 (87.3)	<0.0001
Knew everyone at risk for LF	214 (87.6)	397 (62.3)	<0.0001

a Com-MDA+LM areas received community-based pre-MDA LF education and a community-based lymphedema management program.

b Com-MDA areas received community-based pre-MDA LF education.

c A participant was considered as having knowledge of lymphedema management if the participant knew about at least one of the following: leg exercises, leg washing, or leg elevation.

d P-values derived from the Rao-Scott Likelihood Ratio Test.

## Discussion

The evaluation of the December 2007 MDA in Orissa led to the description of several predictors of and barriers to compliance with the MDA program. These predictors and barriers were used to refine a pre-MDA community-based educational campaign that was then implemented in six blocks in Khurda District, three of which had received the early version of the campaign―the Com-MDA+LM area―and three of which were new to the campaign―the Com-MDA area. The results were remarkable. In the Com-MDA+LM areas MDA compliance increased from a 2007 baseline of 59.5% [16] to 90.2%, well above that target of 80.0% compliance among the entire population. There was also a marked increase in compliance in the Com-MDA areas to 75.0%, which is close to the target and much improved from the 2007 baseline of 52.2% [16]. It is likely that the baseline MDA compliance for rural areas in this district is around 52%, and it is clear that both intervention groups had a significant increase in adherence over that baseline.

This study not only makes an important and direct contribution to the effort to interrupt the transmission of LF in India, it also serves as an example that can be used by other programs to overcome barriers to MDA compliance in affected populations. The KAP survey allowed identification of predictors of and barriers to adherence to a DEC regimen Factors identified in the previous evaluation were targeted by an educational campaign delivered one month prior to the 2008 MDA. The increased adherence during the 2008 MDA campaign provided not only the proof-of-concept that the targeted educational program worked, but it also validated the previously identified predictors and barriers. This assessment demonstrates how critical operational research is to any health program, particularly one whose success depends on changing health behaviors. Fortunately, this research can lead to simple and effective solutions. Developing messages that address key concepts for improving compliance with the MDA program is essential. In Orissa, these include: 1) making people aware of the occurrence of the MDA in advance of its occurrence―the CASA program is launched one month prior to the MDA, 2) making people aware of the purpose of the MDA medication, 3) making people aware that everyone is at risk for infection, 4) making people aware that one can be infected and still feel well, and 5) making people aware that side effects of DEC are infrequent and mild. Additionally, data from those who did not take DEC suggested that the medication's benefit needs to be personalized. The person who takes the medication needs to feel that they or a close family member stands to benefit directly. Lofty national goals did not speak to those who did not take DEC in this evaluation population. Individualized programs will need to be developed to address the specific needs of each location.

One unique and important finding from the 2008 evaluation is that community-based lymphedema management programs positively affect MDA compliance independently of such programs' effects on the other predictors of compliance. Even after multivariable modeling controlling for all of the other LF and MDA knowledge predictors, knowing any one of the three components of the management of leg lymphedema predicted adherence to the DEC regimen. This positive impact of community-based lymphedema management education persisted even among those who had no household members with lymphedema. Additionally, the Com-MDA+LM area had the highest level of persons adhering to the DEC regimen in this study (90.2%). Admittedly, part of the explanation may be that the Com-MDA+LM had received a pre-MDA educational campaign two years in a row, but the campaign in the year 2007, which did not focus on predictors of adherence, was largely ineffectual (as evidenced by DEC adherence of 59.5% in the area in 2007). Previous authors have suggested that morbidity control programs could improve MDA compliance [3], [26], but this study is the first to provide data wholly consistent with, if not unequivocally substantiating, that hypothesis. Perhaps these programs are effective because they help maintain awareness of LF and its chronic manifestations in the community and reinforce LF messages taught in the pre-MDA programs. Or perhaps they enhance trust at the community and individual level by providing programs benefiting a generally marginalized and stigmatized population, those who suffer from lymphedema and elephantiasis. Lymphedema management programs could provide an ideal platform for both LF and MDA education to improve MDA program compliance. As India approaches LF elimination, there will be a continued need to assist LF patients with clinical disease. Integrating lymphedema management with LF elimination efforts could be a more cost-effective way to ensure that MDA compliance remains high, even if political pressure to continue funding elimination efforts diminishes.

There are several factors that could influence compliance that merit further comment. Persons in the Com-MDA+LM areas had the fewest number of people who reported no education and the highest number who reported reading well. In univariable analysis reading well influenced the decision to take DEC and education level had relatively little impact on the decision; in multivariable analysis the relationship reversed. Possibly the ability to weigh the risks and benefits of MDA compliance is more directly related to education level than to literacy. Additionally, the mechanisms utilized to distribute the educational message included many verbal routes (i.e. street plays, auto-rickshaws, etc). However, an assessment of literacy, or health literacy, using a validated tool might allow a more thorough examination of this complex relationship. Multivariable analysis suggested that those with less education were more likely to comply. Perhaps those with less education are more likely to accept public health messages. It is important to note that although the relationship with education level is statistically significant, because of smaller numbers in each group the confidence intervals around the ORs are wide and in many cases approach one. It may be that the impact of education on compliance is much less than suggested by our analysis; this is an issue that should certainly be examined in future studies.

Knowing a household member with leg edema could also influence one's perception of risk for LF. While the prevalence of leg edema in a household member did not differ statistically across the three groups, the highest prevalence was reported in the Com-MDA+LM group. Whether this represents actual increased prevalence or increased recognition of the condition because of the lymphedema management program is unclear. Even though this factor was found to be a predictor of MDA compliance in univariable analysis, it was not significant in multivariable analysis. One possible reason for this is that the CASA educational message emphasized that everyone was at risk for infection and that one might be infected even if one felt well.

Finally, there was an interaction between knowing about the MDA in advance and knowing everyone was at risk for LF. Those who only knew everyone was at risk for LF had a small increase in MDA compliance. Those who only knew about the MDA in advance had a larger increase. Those who knew both had a synergistically larger increase. Why this was so is not clear. Perhaps those who understood both messages had a heightened sense of benefit or felt more empowered to achieve their own health goals because they felt at risk for infection and that they had the opportunity to avail themselves of preventive medication. Perhaps the interaction reflects the influence of another factor, such as an understanding of side effects and their management. In either case, the interaction points to the importance of addressing risk of LF and opportunity to access the beneficial MDA medication in any educational message.

The limitations of this evaluation are similar to other retrospective evaluations that use the EPI random walk cluster method. Selection bias was reduced by defining a strict set of rules governing household selection and replacement of non-participants was not allowed. The evaluation was cross-sectional, so causality cannot be assumed. However, given that most of the predictors identified in this evaluation were the same as in the prior evaluation and that the knowledge of the predictors in the Com-MDA+LM area was higher in this evaluation that in the prior one, it is likely that the predictors are causal. The generalizability of the results may be limited to rural areas as urban areas were not included. Finally, although there is a definitive baseline MDA compliance for the Com-MDA+LM area, the baseline for the Com-MDA and the MDA-only areas are based on less direct empirical data. The Com-MDA baseline is derived from the Bologarh MDA compliance of 52.2% for the 2007 MDA. The fact that the compliance in Banapur for the 2008 MDA was 52.9% suggests that MDA compliance in rural areas of Khurda District is approximately 50–55%.

Determining the predictors and barriers of adherence to the DEC regimen distributed in the MDA allowed for identification of key educational messages that were incorporated into a pre-MDA community-based LF educational campaign and resulted in a marked increase in regimen adherence. An added benefit was demonstrating that community-based lymphedema management programs independently enhanced adherence. Although further work is needed to determine exactly which components of lymphedema management programs influence MDA program compliance, one should not wait for those results before investing in such programs which address the twin goals of improving the lives of those suffering from filarial disease and increasing compliance with MDA programs to the level needed for the interruption of LF transmission.

## Supporting Information

Checklist S1 STROBE checklist.
(0.08 MB DOC)
